# The Post-Lockdown Era: What Is Next in Italy?

**DOI:** 10.3389/fphar.2020.01074

**Published:** 2020-07-15

**Authors:** Cristoforo Pomara, Giovanni Li Volti, Francesco Cappello

**Affiliations:** ^1^ Department of Medical, Surgical Sciences and Advanced Technologies “G.F. Ingrassia,” University of Catania, Catania, Italy; ^2^ Medico-Legal Unit, University Hospital “Policlinico Vittorio Emanuele,” Catania, Italy; ^3^ Department of Biomedical and Biotechnological Sciences, University of Catania, Catania, Italy; ^4^ Department of Biomedicine, Neuroscience and Advanced Diagnostics, University of Palermo, Palermo, Italy; ^5^ Department of Biology, Temple University, Philadelphia, PA, United States

**Keywords:** COVID-19, infectious diseases, autopsy, diagnosis, mortality

The current outbreak of the COVID-19 infection, which started in December 2019 in Wuhan (China), was declared a pandemic by the World Health Organization (WHO) on the 11^th^ of March, 2020.

On the 14^th^ of March, Remuzzi A. and Remuzzi G. published a very interesting article describing a prediction model to prepare Italian political leaders for the potential spread of COVID-19 in Italy and to improve the capacity of the national health system (Remuzzi and Remuzzi, 2020).

One month later, the scenario seems to have completely changed: while most patients having COVID-19 pneumonia can have a mild disease course, some patients could develop severe respiratory distress, and frequently coagulopathy, generating acute pulmonary embolism caused by *in-situ* thrombosis due to interstitial COVID-19 injury (Klok et al., 2020; Messina et al., 2020; Rotzinger et al., 2020).

In our opinion, in order to better contain the outbreak, a broader effort is required from our leaders to help physicians fighting this challenge, not only to improve the capacity of our national health system, but also to prevent death by the use of proper treatments, allowing physicians to learn from the dead.

To the best of our knowledge, to date, autopsy findings were briefly reported in only a few papers, describing several important aspects of the COVID-19 outbreak (Barton et al., 2020; Cai et al., 2020; Carsana et al., 2020; Karami et al., 2020; Magro et al., 2020; Tian et al., 2020a; Tian et al., 2020b; Wichmann et al., 2020; Xu et al., 2020). Other papers can be read only in a pre-print version, considering that they are still under review.

There is therefore a lack of crucial information that cannot be appreciated by means other than direct examination and histopathological data collection (Mao et al., 2020), resulting in not verified post-mortem diagnoses.

Several retrospective studies tried to identify the risk factors associated with death in COVID-19 patients, but the available data can only suggest a potential involvement of specific organs (Wu et al., 2020; Yang et al., 2020; Zhou et al., 2020).

Many physicians are wondering if we are not facing a systemic pathology that affects the vessels of different anatomical districts, not only the lung, but the heart, kidney, liver, intestine, brain, and even the skin. This hypothesis is supported by the fact that angiotensin-converting enzyme 2 (ACE2), and putatively also sialic acids, the suggested “doors” by which COVID-19 could enter endothelial cells and pericytes, are almost ubiquitarian, and not only present in the endothelial cells of alveolar membranes (Letko et al., 2020; Shang et al., 2020).

In addition, molecular mimicry has been proposed as a mechanism inducing autoimmune damage at the endothelial level (Cappello, 2020), but the lack of histological specimens has hindered the possibility to test this hypothesis.

Collecting cadavers’ samples or biological fluids and swabs can also be useful in the control of epidemics, as shown during previous infectious disease outbreaks. It is a fact that, during the West Africa Ebola epidemic, in the Ebola Virus Disease (EVD) surveillance strategy, RNA virus was isolated in body fluids days or months after the onset of the disease from any living or deceased individual who had, or had had, clinical symptoms compatible with EVD. Thanks to this procedure, it was possible to monitor the number of infected patients in order to recognize new sources of transmission and to control the epidemic phenomenon (Kreuels et al., 2014; Kreuels et al., 2015; Petrosillo et al., 2015; Varkey et al., 2015; Castillo et al., 2016; Vetter et al., 2016). Without performing systematically autopsies on all patients who die of (or with) COVID-19 disease, we will never find the end of the skein.

In view of what has been stated, we also want to denote raise a political issue: the WHO suggested performing post-mortem examinations for those who die of/with COVID-19 following recommended safety procedures (WHO Interm Guidance, 2020). Although the first data about the autopsy findings suggested the pivotal role of autopsy in the management of unknown disease, many governments—including the Italian one—did not make sufficient efforts to permit forensic doctors and gross pathologists to finally perform autopsies on these corpses (Pomara et al., 2020; Salerno et al., 2020). In Italy, in the first phase of the outbreak, the Ministry of Health with a specific act (Circular of General Direction of Health Prevention) discouraged the use of autopsies for COVID-19 deaths (Italian Ministry of Health, 2020a): indeed, to date, only one study has been published about the Italian experience in the COVID-19 post-mortem examination. Carsana et al. described the histological examination of lung tissue samples from 38 consecutive patients who died from COVID-19 between the 29^th^ of February and the 24^th^ of March (Carsana et al., 2020). Considering that in Italy more than 30,000 people have died from or with COVID-19, it is possible to confirm that almost no autopsies have been performed. The 1^st^ of June the Italian Ministry of Health revised its act (Italian Ministry of Health, 2020b) by authorizing autopsies on people who died for/with COVID-19, but in our opinion—due to the current epidemic data in Italy—it was a big mistake and a missed opportunity to not have done this sooner.

We cannot, though, underestimate the importance of autopsy as a diagnostic tool to understand the underlying mechanisms behind death. In accordance with the WHO, post-mortem examination for deceased persons infected with COVID-19 should be consistent with those used for any autopsies of people who have died from an acute respiratory illness, following the recommended safety procedures.

Political and health authorities should therefore be aware that performing systematic autopsies on patients who die following COVID-19 infection can provide significant responses concerning the real mechanisms underlying virus related deaths and organ injury. Encouraging autopsy practice as an investigative tool could also help physicians to define effective treatment to reduce mortality. Another essential aspect is related to “COVID-19 survivor” management. In our opinion, a COVID-19 survivor is a person who has been infected by the virus with a hospitalization period in the intensive care unit, who is still living. When a person becomes a survivor after completing treatment, several issues should be discussed: what are the consequences of the infection? Which are the organs that have been damaged? Which are the organs involved? The management of the COVID-19 survivor is the next challenge for the scientific community. Furthermore, other severe diseases should be considered for COVID-19 survivors such as depression and anxiety in order to provide them the best possible medical care to improve their quality of life.

As the authors reported, measures are urgently needed to avoid unnecessary deaths and we suggest that autopsy is an important tool to learn from inevitable deaths. Moreover, improving our knowledge about the COVID-19 infection, may provide useful insights in order to establish, as soon as possible, the proper guidelines for the medical management of COVID-19 survivors.

## Author Contributions

Conceptualization: CP, GV, and FC. Writing—original draft preparation: CP, GV, and FC. Writing—review and editing: CP, GV, and FC. All authors contributed to the article and approved the submitted version.

## Conflict of Interest

The authors declare that the research was conducted in the absence of any commercial or financial relationships that could be construed as a potential conflict of interest.

## References

[B1] Barton L. M. Duval E. J. Stroberg E. Ghosh S. Mukhopadhyay S. (2020). COVID-19 Autopsies, Oklahoma, USA. Am. J. Clin. Pathol. 153, 725–733. 10.1093/ajcp/aqaa062 32275742 PMC7184436

[B2] Cai Y. Hao Z. Gao Y. Ping W. Wang Q. Peng S. (2020). Coronavirus Disease 2019 in the Perioperative Period of Lung Resection: A Brief Report From a Single Thoracic Surgery Department in Wuhan, People’s Republic of China. J. Thorac. Oncol. 15, 1065–1072. 10.1016/j.jtho.2020.04.003 32289516 PMC7194109

[B3] Cappello F. (2020). Is COVID-19 a proteiform disease inducing also molecular mimicry phenomena? Cell Stress Chaperones. 10.1007/s12192-020-01112-1 PMC7167495 32314313

[B4] Carsana L. Sonzogni A. Nasr A. Rossi R. S. Pellegrinelli A. Zerbi P. (2020). Pulmonary post-mortem findings in a series of COVID-19 cases from northern Italy: a two-centre descriptive study. Lancet Infect. Dis. . 10.1016/S1473-3099(20)30434-5 PMC7279758 32526193

[B5] Castillo P. Martínez M. J. Ussene E. Jordao D. Lovane L. Ismail M. R. (2016). Validity of a Minimally Invasive Autopsy for Cause of Death Determination in Adults in Mozambique: An Observational Study. PloS Med. 13 (11), e1002171. 10.1371/journal.pmed.1002171 27875530 PMC5119723

[B6] Italian Ministry of Health (2020a). Emergency indications related to the COVID-19 epidemic concerning the funeral sector, cemetery, and cremation. Circular of General Direction of Health Prevention; 2020. Available online: http://www.trovanorme.salute.gov.it/norme/renderNormsanPdf?anno=2020&codLeg=73965&parte=1%20&serie=null (accessed May 3, 2020).

[B7] Italian Ministry of Health (2020b). Emergency Indications Related to the COVID-19 Epidemic Concerning the Funeral Sector, Cemetery, and Cremation - Update following the new legislative indications and epidemiological data. Circular of General Direction of Health Prevention; 2020. Available online: https://opi.roma.it/archivio_news/news/1949/5740%20Aggiornamento%20Circolare%20serv%20funebri%2028_5_2020.pdf (accessed May 31, 2020).

[B8] Karami P. Naghavi M. Feyzi A. Aghamohammadi M. Novin M. S. Mobaien A. (2020). Mortality of a pregnant patient diagnosed with COVID-19: A case report with clinical, radiological, and histopathological findings. Travel Med. Infect. Dis. 101665. 10.1016/j.tmaid.2020.101665 32283217 PMC7151464

[B9] Klok F. A. Kruip M. J. H. A. van der Meer N. J. M. Arbous M. S. Gommers D. A. M. P. J. Kant K. M. (2020). Incidence of thrombotic complications in critically ill ICU patients with COVID-19. Thromb. Res. 191, 145–147. 10.1016/j.thromres.2020.04.013 32291094 PMC7146714

[B10] Kreuels B. Wichmann D. Emmerich P. Schmidt-Chanasit J. De Heer G. Kluge S. (2014). A case of severe Ebola virus infection complicated by gram-negative septicemia. N. Engl. J. Med. 371, 2394–2401. 10.1056/NEJMoa1411677 25337633

[B11] Kreuels B. Addo M. M. Schmiedel S. (2015). Severe Ebola virus infection complicated by gram-negative septicemia. N. Engl. J. Med. 372 (14), 1377. 10.1056/NEJMc1500455 25830439

[B12] Letko M. Marzi A. Munster V. (2020). Functional assessment of cell entry and receptor usage for SARS-CoV-2 and other lineage B betacoronaviruses. Nat. Microbiol. 5, 562–569. 10.1038/s41564-020-0688-y 32094589 PMC7095430

[B13] Magro C. Mulvey J. J. Berlin D. Nuovo G. Salvatore S. Harp J. (2020). Complement associated microvascular injury and thrombosis in the pathogenesis of severe COVID-19 infection: A report of five cases. Transl. Res. 220, 1–13. 10.1016/j.trsl.2020.04.007 32299776 PMC7158248

[B14] Mao R. Liang J. Shen J. Ghosh S. Zhu L.-R. Yang H. (2020). Implications of COVID-19 for patients with pre-existing digestive diseases. Lancet Gastroenterol. Hepatol. 5 (5), 425–427. 10.1016/S2468-1253(20)30076-5 32171057 PMC7103943

[B15] Messina G. Polito R. Monda V. Cipolloni L. Di Nunno N. Di Mizio G. (2020). Functional Role of Dietary Intervention to Improve the Outcome of COVID-19: A Hypothesis of Work. Int. J. Mol. Sci. 21 (9), 3104. 10.3390/ijms21093104 PMC7247152 32354030

[B16] Petrosillo N. Nicastri E. Lanini S. Capobianchi M. R. Di Caro A. Antonini M. (2015). Ebola virus disease complicated with viral interstitial pneumonia: A case report. BMC Infect. Dis. 15, 432. 10.1186/s12879-015-1169-4 PMC4608352 26471197

[B17] Pomara C. Volti G. L. Cappello F. (2020). COVID-19 Deaths: Are We Sure It Is Pneumonia? Please, Autopsy, Autopsy, Autopsy! J. Clin. Med. 9, 1259. 10.3390/JCM9051259 PMC7287760 32357503

[B18] Remuzzi A. Remuzzi G. (2020). COVID-19 and Italy: what next? Lancet. 10.1016/S0140-6736(20)30627-9 PMC7102589 32178769

[B19] Rotzinger D. C. Beigelman-Aubry C. von Garnier C. Qanadli S. D. (2020). Pulmonary embolism in patients with COVID-19: Time to change the paradigm of computed tomography. Thromb. Res. 190, 58–59. 10.1016/j.thromres.2020.04.011 32302782 PMC7151364

[B20] Salerno M. Sessa F. Piscopo A. Montana A. Torrisi M. Patanè F. (2020). No Autopsies on COVID-19 Deaths: A Missed Opportunity and the Lockdown of Science. J. Clin. Med. 9 (5), 1472. 10.3390/jcm9051472 PMC7291342 32422983

[B21] Shang J. Ye G. Shi K. Wan Y. Luo C. Aihara H. (2020). Structural basis of receptor recognition by SARS-CoV-2. Nature 581 (7807), 221–224. 10.1038/s41586-020-2179-y 32225175 PMC7328981

[B22] Tian S. Hu W. Niu L. Liu H. Xu H. Xiao S.-Y. (2020a). Pulmonary pathology of early phase 2019 novel coronavirus (COVID-19) pneumonia in two patients with lung cancer. J. Thorac. Oncol. 15 (5), 700–704. 10.1016/j.jtho.2020.02.010 32114094 PMC7128866

[B23] Tian S. Xiong Y. Liu H. Niu L. Guo J. Liao M. (2020b). Pathological study of the 2019 novel coronavirus disease (COVID-19) through postmortem core biopsies. Mod. Pathol. 33 (6), 1007–1014. 10.1038/s41379-020-0536-x 32291399 PMC7156231

[B24] Varkey J. B. Shantha J. G. Crozier I. Kraft C. S. Lyon G. M. Mehta A. K. (2015). Persistence of ebola virus in ocular fluid during convalescence. N. Engl. J. Med. 372 (25), 2423–2427. 10.1056/NEJMoa1500306 25950269 PMC4547451

[B25] Vetter P. Fischer W. A. Schibler M. Jacobs M. Bausch D. G. Kaiser L. (2016). Ebola Virus Shedding and Transmission: Review of Current Evidence. J. Infect. Dis. 214 (suppl 3), S177–S184. 10.1093/infdis/jiw254 27443613 PMC6283352

[B26] WHO Interm Guidance (2020). Infection Prevention and Control for the safe management of a dead body in the context of COVID-19. J. Hosp. Infect. 104, 246–251. 10.1016/j.jhin.2020.01.022 32035997 PMC7132493

[B27] Wichmann D. Sperhake J.-P. Lütgehetmann M. Steurer S. Edler C. Heinemann A. (2020). Autopsy Findings and Venous Thromboembolism in Patients With COVID-19. Ann. Intern. Med. 10.7326/M20-2003 PMC7240772 32374815

[B28] Wu C. Chen X. Cai Y. Xia J. Zhou X. Xu S. (2020). Risk Factors Associated With Acute Respiratory Distress Syndrome and Death in Patients With Coronavirus Disease 2019 Pneumonia in Wuhan, China. JAMA Intern. Med. 180 (7), 1–11. 10.1001/jamainternmed.2020.0994 PMC7070509 32167524

[B29] Xu Z. Shi L. Wang Y. Zhang J. Huang L. Zhang C. (2020). Pathological findings of COVID-19 associated with acute respiratory distress syndrome. Lancet Respir. Med. 8 (4), 420–422. 10.1016/S2213-2600(20)30076-X 32085846 PMC7164771

[B30] Yang X. Yu Y. Xu J. Shu H. Xia J. Liu H. (2020). Clinical course and outcomes of critically ill patients with SARS-CoV-2 pneumonia in Wuhan, China: a single-centered, retrospective, observational study. Lancet Respir. Med. 8 (5), 475–481. 10.1016/S2213-2600(20)30079-5 32105632 PMC7102538

[B31] Zhou F. Yu T. Du R. Fan G. Liu Y. Liu Z. (2020). Clinical course and risk factors for mortality of adult inpatients with COVID-19 in Wuhan, China: a retrospective cohort study. Lancet 395, 1054–1062. 10.1016/S0140-6736(20)30566-3 32171076 PMC7270627

